# Isolation and Purification of a Neuroprotective Phlorotannin from the Marine Algae *Ecklonia maxima* by Size Exclusion and High-Speed Counter-Current Chromatography

**DOI:** 10.3390/md17040212

**Published:** 2019-04-04

**Authors:** Xuezhen Zhou, Mengqi Yi, Lijian Ding, Shan He, Xiaojun Yan

**Affiliations:** Li Dak Sum Yip Yio Chin Kenneth Li Marine Biopharmaceutical Research Center, College of Food and Pharmaceutical Sciences, Ningbo University, Ningbo 315800, China; zhouxuezhen@nbu.edu.cn (X.Z.); ymqnbu@163.com (M.Y.); heshan@nbu.edu.cn (S.H.)

**Keywords:** phlorotannin, eckmaxol, high-speed counter-current chromatography, NMR spectroscopy, mass spectrometry, isolation and purification, *Ecklonia maxima*

## Abstract

Phlorotannins are polyphenolic metabolites of marine brown algae that have been shown to possess health-beneficial biological activities. An efficient approach using a combination of high-speed counter-current chromatography (HSCCC) and size exclusion chromatography with a Sephadex LH-20 has been successfully developed for the isolation and purification of a neuroprotective phlorotannin, eckmaxol, from leaves of the marine brown algae, *Ecklonia maxima*. The phlorotannin of interest, eckmaxol, was isolated with purity >95% by HSCCC using an optimized solvent system composed of *n*-hexane–ethyl acetate–methanol–water (2:8:3:7, v/v/v/v) after Sephadex LH-20 size exclusion chromatography. This compound was successfully purified in the quantity of 5.2 mg from 0.3 kg of the *E. maxima* crude organic extract. The structure of eckmaxol was identified and assigned by NMR spectroscopic and mass spectrometric analyses. The purification method developed for eckmaxol will facilitate the further investigation and development of this neuroprotective agent as a drug lead or pharmacological probe. Furthermore, it is suggested that the combination of HSCCC and size exclusion chromatography could be more widely applied for the isolation and purification of phlorotannins from marine algae.

## 1. Introduction

**Figure 1:**
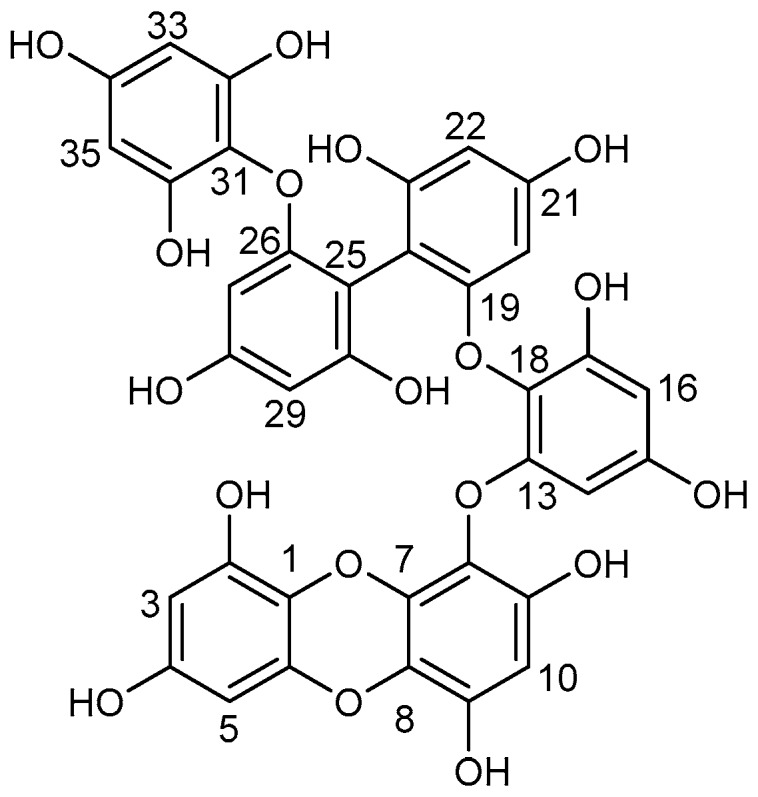
Chemical structure of eckmaxol (**1**).

Phlorotannins are the major phloroglucinol-derived polyphenols of wide occurrence among marine brown algae, and these have been extensively investigated for various biological activities such as antioxidant, anticancer, anti-inflammatory, anti-allergic, antidiabetic, antihypertensive, and neuroprotective effects [1,2,3,4]. *Ecklonia maxima* is a brown seaweed that is distributed along the west coast of South Africa [5]. The leaves of *E. maxima* are frequently used as source materials for producing alginate, animal feed, nutritional supplements, fertilizers, and for preparation of different medications, including alpha-glucosidase inhibitors applied for the treatment of diabetes mellitus [5,6]. Eckmaxol (**1**; Figure 1) is a recently reported phlorotannin obtained from the ethyl acetate extract of *E. maxima* as described in our previous study. This phlorotannin possesses many beneficial properties, including protection against Aβ oligomer induced neurotoxicity in SH-SY5Y cells in vitro [7]. Additional material is required to further investigate the functions of **1** in mammalian systems for the development of this compound as a new pharmacological probe or potential drug lead for treating Alzheimer’s disease. 

Unfortunately, the reported purification of phlorotannins has typically included repeated column chromatography steps and preparative HPLC, which is time-consuming, leads to the loss of target phlorotannins due to oxidization during the long process, and is not industrially viable due to the cost of solid supports (e.g., resin or gel) for separation. Accordingly, these techniques are not typically suitable for purification of large quantities of material unless no other methods can complete the task [8,9]. Alternatively, high-speed counter-current chromatography (HSCCC) is a liquid-liquid separation chromatography that can reduce the separation time and cost, and offers effectively total sample recovery due to the lack of a solid support matrix that can degrade or permanently retain target molecules [10]. HSCCC has recently been applied to the isolation of various natural products, most typically coming from plants [11,12,13]. However, no previous report has disclosed the use of HSCCC for the isolation and purification of phlorotannins. The purpose of this study was to develop an efficient method for the preparative isolation and purification of eckmaxol (**1**) using the combined methods HSCCC and Sephadex LH-20, which are both nondestructive and nonabsorptive (e.g., lossless) techniques.

## 2. Results and Discussion

### 2.1. Enrichment of Eckmaxol by Sephadex LH-20 Size Exclusion Chromatography

Due to the large amounts of pigments and other unknown polyphenols contained in the crude ethanolic extract of *E. maxima*, it is difficult to separate and isolate **1** with high purity by HSCCC in one step. In order to effectively enrich the targeted compound, the crude extract was first subjected to size exclusion chromatography on a Sephadex LH-20 gravity column. The LH-20 column was eluted with an isocratic solvent system of dichloromethane–methanol (1:1). A total of six fractions (250 mL each) were successively collected, named Fractions A–F. Fr. C was determined to contain **1** in high quantity, and this sample was concentrated to dryness and stored in a refrigerator (4 °C) for later HSCCC separation.

### 2.2. Optimization of UPLC Analysis for Eckmaxol 

**Figure 2:**
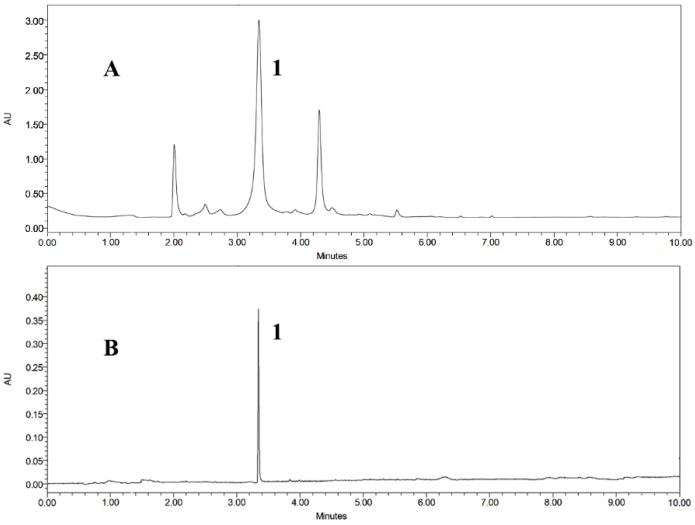
Representative ultra-performance liquid chromatography (UPLC) chromatograms (254 nm) of samples prepared from *E. maxima* (**A**). Fraction C from the size exclusion chromatography of the crude ethyl acetate extract; (**B**) Subfraction of C that contains **1** after preparative separation by high-speed counter-current chromatography (HSCCC).

A ultra-performance liquid chromatography (UPLC) method was developed to ensure the baseline separation of the target compound and impurities, and evaluate the size exclusion chromatography fraction C. Different flow rates, elution modes, detection wavelengths and column temperatures were screened. The result indicated that the target compound was baseline separated with methanol-water (methanol: 0–10 min, 10%–90%) as the solvent system, when the flow rate, column temperature and detection wavelength were set at 0.4 mL/min, 25 °C and 254 nm. Preliminary assignment of **1** in the chromatogram was made by comparison of peak retention time and UV spectrum against a previously derived authentic standard. The UPLC chromatogram of fraction C showed the major, but not only peak, as being **1** (Figure 2A).

### 2.3. Selection of the HSCCC Two-Phase Solvent System

Since HSCCC relies on two immiscible liquids to function as stationary and mobile phases, the selection of a suitable biphasic solvent system plays a vital role in successful separations. It has been suggested that the partition coefficient (K) is the most important parameter in solvent system selection, which should be 0.5 ≤ K ≤ 2 (close to 1, best) to get a good separation for HSCCC in a suitable run time [13,14]. As previously reported in the literature [15,16], the two-phase solvent system “HEMWat”, comprising *n*-hexane–ethyl acetate–methanol–water, has been widely applied in the separation of natural products by HSCCC. Five sets of different proportional two-phase HEMWat solvent systems were carried out to determine the partition value, K, of the target compound at various volume ratios of n-hexane/ethyl acetate/methanol/water (3:10:3:10, 1:3:1:3, 2:7:3:7, 2:8:3:7, all v/v/v/v) by UPLC analysis of each partition. The results, shown in Table 1, indicated that the two-phase solvent system of 2:8:3:7 *n*-hexane/ethyl acetate/methanol/water, v/v/v/v, provided a suitable partition value for eckmaxol of *K* = 1.15 with good resolution and short elution time.

### 2.4. HSCCC Separation

**Figure 3:**
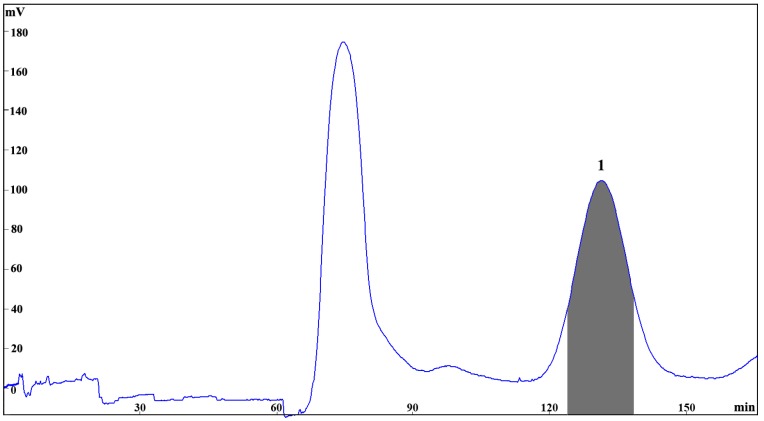
HSCCC chromatogram of the fraction C of the ethanol extract from *E. maxima* using the two-phase solvent system composed of *n*-hexane–EtOAc–MeOH–water (2:8:3:7, *v/v/v/v*); stationary phase: upper phase of solvent system; mobile phase: lower aqueous phase of solvent system; column capacity 320 mL; rotation speed 850 rpm; column temperature 25 °C; flow rate 2.0 mL/min; detection, 254 nm; sample injected, 300 mg in 6 mL biphasic solution; retention of the stationary phase, 65.7%; peak identification: eckmaxol (**1**).

The selected fraction C from the size exclusion chromatography of the ethanol extract from *E. maxima* (300 mg) was applied for HSCCC separation with the chosen two-phase solvent system, *n*-hexane-ethyl acetate–methanol–water (2:8:3:7). In order to optimize the resolution and reduce the separation time, different flow rates and rotation speeds were evaluated. It was found that when the flow rate was 2 mL/min and rotation speed was 850 rpm, a good separation was achieved for elution of **1** with a good stationary phase retention of 65.7%. The HSCCC peak fraction corresponding to **1** (5.2 mg) was collected and determined to have purity of 95.83% by UPLC analysis (Figure 2B). The resulting HSCC chromatogram is shown in Figure 3, demonstrating the good resolution and peak shape of compound **1** at *t*_R_ = 140 min.

### 2.5. Identification of Chemical Structure

Compound **1** was identified by HR-ESI-MS, ^1^H-NMR, and ^13^C-NMR after purification by HSCCC, and its detailed data are shown in Table 2. Its molecular formula C_36_H_24_O_18_ was deduced by HR-ESI-MS data at *m/z* 743.0896 [M – H]^–^. Compound **1** was identified as a phlorotannin, eckmaxol, with the chemical structure as shown in Figure 1. The structure of eckmaxol was first disclosed in a Japanese patent application (JP 2013-49639), but the assignment of its spectroscopic data was never reported.

## 3. Materials and Methods 

### 3.1. Reagents and Materials

All solvents used for HSCCC were of analytical grade (Huadong Chemicals, Hangzhou, China). Reverse osmosis Milli-Q water (18 M) (Millipore, Bedford, MA, USA) was used for all solutions and dilutions. Methanol used for UPLC analyses was of chromatographic grade and purchased from Anpel Laboratory Technologies (Shanghai, China). The CD_3_OD used for NMR analyses was purchased from Tenglong Weibo Technology (Qingdao, China). The sample of *E. maxima* (no. 801) was kindly provided by Shandong Jiejing Group Co., Ltd. in China, collected from the seashore of South Africa.

### 3.2. Apparatus 

HSCCC was carried out using a model TBE-300C high-speed countercurrent chromatograph (Tauto Biotech Co. Ltd., Shanghai, China), containing a self-balancing three-coil centrifuge rotor equipped with three preparative multilayer coils and a total capacity of 320 mL. The internal diameter of PTFE (Polytetrafluoroethylene) tubing was 1.9 mm. The apparatus maximum rotational speed is 1000 rpm and has a 20 mL manual sample loop. The revolution radius was 5 cm and the value of the multilayer coil varied from 0.5 at the internal terminal to 0.8 at the external terminal. An integrated TBP 5002 (Tauto Biotech Co. Ltd.) was used to pump the two-phase HSCCC solvent system, and the UV absorbance of the effluent was measured at 254 nm by a UV 1001 detector (Shanghai Sanotac Scientific Instruments Co. Ltd., Shanghai, China). A DC-0506 constant temperature regulator (Tauto Biotech Co. Ltd.) was used to control the temperature during HSCCC. An N2000 data analysis system (Institute of Automation Engineering, Zhejiang University, Hangzhou, China) was employed for HSCCC data collection and analysis. The UPLC equipment was using a Waters Acquity a UPLC BEH C18 column (100 mm × 2.1 mm, 1.7 μm particle size) equipped with a model 2998 diode array detector and Empower System (Waters Co., Milford, MA, USA). NMR experiments including ^1^H, ^13^C, DEPT, ^1^H-^1^H COSY, HSQC, and HMBC were carried out using a Varian 500 MHz NMR spectrometer (Palo Alto, CA, USA) spectrometer. HR-ESI-MS data was measured using a Waters Q-TOF Premier LC/MS spectrometer (Waters Co., Milford, MA, USA). Column chromatography (CC) was carried out with Sephadex LH-20 (Amersham Biosciences, Piscataway, NJ, USA). 

### 3.3. Preparation of Crude Sample from E. maxima for HSCCC

The leaves of *E. maxima* were cut into pieces. The fresh pieces (~0.3 kg, wet) were extracted three times with 1 L of 80% ethanol (EtOH/H_2_O) for 1.5 h by sonication at room temperature (25 °C). The crude extract was concentrated in a rotary vacuum evaporator and partitioned. Then the dried EtOAc extract (1 g) was subjected to column chromatography with Sephadex LH-20 gel for fractionation to furnish fractions A–F, each eluted with a mixed solvent system of dichloromethane–methanol (1:1). Fr. C was concentrated to dryness and stored in a refrigerator (4 °C) for later HSCCC separation.

### 3.4. Preparation of Two-Phase Solvent System and Sample Solution

The HSCCC experiments were performed using a two-phase solvent system comprising *n*-hexane/ethyl acetate/methanol/water (2:8:3:7, v/v/v/v) solvent. The two phases were separated after thoroughly equilibrating the mixture in a separating funnel at 25 °C. The upper organic phase was used as the stationary phase, and the lower aqueous phase was employed as the mobile phase.

### 3.5. HSCCC Separation

The HSCCC column was initially filled with the organic stationary phase and rotated at 850 rpm; the mobile phase was pumped into the column in the descending mode at the same flow rate used for separation (2 mL/min). When the mobile phase emerged from the column, it indicated that hydrodynamic equilibrium had been achieved. The concentrated fraction C (300 mg) obtained from the 80% EtOH extract of *E. maxima* was dissolved in 6 mL of a 1:1 (v/v) mixture of the two HSCCC solvent system phases and injected to the sample port. The effluent from the HSCCC was monitored by UV at 254 nm, and 6 mL fractions were collected in 8 mL tubes by a fraction collector.

### 3.6. Analysis and Identification of the Target Compound

The fraction generated by preparative HSCCC was evaluated by UPLC. The sample was separated with a CH_3_OH/H_2_O gradient (flow 0.4 mL/min, 10%–90% CH_3_OH from 0–10 min). The effluent was continuously monitored by a UV detector at 254 nm. The fraction that showed only one peak in the chromatogram was respectively pooled together to yield the compound (5.2 mg, *t*_R_ 3.3 min).

## 4. Conclusions

In conclusion, an efficient method relying on HSCCC after size exclusion chromatography on Sephadex LH-20 was used to preparative separation of eckmaxol (**1**) from the leaves of *E. maxima* in a lossless two chromatic step procedure. It was important to preliminarily fractionate the crude extract for HSCCC separation to improve the resolution and efficiency. The solvent system of *n*-hexane/ethyl acetate/methanol/water (2:8:3:7, *v/v/v/v*) was used to isolate eckmaxol (**1**). The separation condition was selected as follow: flow rate 2.0 mL/min, rotary speed 850 rpm, column temperature 25 °C. Under the optimized HSCCC condition, 5.2 mg eckmaxol with the high purity of 95.83% was isolated from 300 mg of fraction C of *E. maxima*. This is the first report of the isolation of eckmaxol (**1**) by integrating HSCCC and size exclusion chromatography, and this method could be used for the effective isolation of different phlorotannins. This convenient and economical approach will be applicable for scale-up production of eckmaxol to increase the yield. The purification method developed for eckmaxol will also facilitate the further investigation and development of this neuroprotective agent as a drug lead or pharmacological probe, ideally through future in vivo studies.

## Figures and Tables

**Table 1:** *K* values of target compound **1** in different ratios of the HEMWat solvent system.

Solvent System	Ratio (v/v/v/v)	*K*
*n*-hexane/ethyl acetate/methanol/water	3:10:3:10	0.32
*n*-hexane/ethyl acetate/methanol/water	1:3:1:3	0.75
*n*-hexane/ethyl acetate/methanol/water	2:7:3:7	1.78
*n*-hexane/ethyl acetate/methanol/water	2:8:3:7	1.15

**Table 2:** ^1^H (500 MHz) and ^13^C-NMR (125 MHz) spectroscopic data of eckmaxol (**1**) in CDOD_3_.

Pos.	*δ*_H_	*δ*_C_	Pos.	*δ*_H_	*δ*_C_
1		124.8	19		157.1
2		151.3	20	5.93 (1H, d, *J* = 2.4 Hz, H-20)	94.2
3	6.11 (1H, d, *J* = 2.4 Hz, H-3)	97.6	21		159.2
4		156.4	22	6.32 (1H, d, *J* = 2.4 Hz, H-22)	98.7
5	5.86 (1H, d, *J* = 2.4 Hz, H-5)	94.7	23		143.3
6		154.4	24		102.0
7		123.5	25		101.8
8		137.7	26		156.1
9		27	143.5	6.20 (1H, d, *J* = 2.0 Hz, H-27)	95.2
10	6.20 (1H, s, H-10)	98.9	28		159.3
11		146.3	29	6.28 (1H, d, *J* = 2.0 Hz, H-29)	98.3
12		124.7	30		158.5
13		153.4	31		124.0
14	6.01 (1H, d, *J* = 2.4 Hz, H-14)	99.3	32		151.6
15		158.7	33	6.03 (1H, s, H-33)	96.1
16	5.94 (1H, d, *J* = 2.4 Hz, H-16)	95.2	34		156.1
17		146.7	35	6.03 (1H, s, H-35)	96.1
18		123.8	36		151.6

## References

[1] Li Y.X., Wijesekara I., Li Y., Kim S.K. (2011). Phlorotannins as bioactive agents from brown algae. Process Biochem..

[2] Kannan R.R.R., Aderogba M.A., Ndhlala A.R., Stirk W.A., Staden J.V. (2013). Acetylcholinesterase inhibitory activity of phlorotannins isolated from the brown alga, *Ecklonia maxima* (Osbeck) Papenfuss. Food Res. Int..

[3] A-Reum K., Tai-Sun S., Min-Sup L., Ji-Young P., Kyoung-Eun P., Na-Young Y., Jong-Soon K., Jae-Sue C., Byeong-Churl J., Dae-Seok B. (2009). Isolation and identification of phlorotannins from *Ecklonia stolonifera* with antioxidant and anti-inflammatory properties. J. Agr. Food Chem..

[4] Thomas N.V., Kim S.K. (2011). Potential pharmacological applications of polyphenolic derivatives from marine brown algae. Environ. Toxicol. Phar..

[5] Rengasamy K.R.R., Aderogba M.A., Amoo S.O., Stirk W.A., Johannes V.S. (2013). Potential antiradical and alpha-glucosidase inhibitors from *Ecklonia maxima* (Osbeck) Papenfuss. Food Chem..

[6] Rengasamy K.R.R., Kulkarni M.G., Stirk W.A., Staden J.V. (2015). Eckol—A new plant growth stimulant from the brown seaweed *Ecklonia maxima*. J. Appl. Phycol..

[7] Wang J., Zheng J., Huang C., Zhao J., Lin J., Zhou X., Naman C.B., Wang N., Gerwick W.H., Wang Q. (2018). Eckmaxol, a phlorotannin extracted from *Ecklonia maxima*, produces anti-*β*-amyloid oligomer neuroprotective effects possibly via directly acting on glycogen synthase kinase 3*β*. ACS Chem. Neurosci..

[8] Shibata T., Ishimaru K., Kawaguchi S., Yoshikawa H., Hama Y. (2008). Antioxidant activities of phlorotannins isolated from Japanese Laminariaceae. J. Appl. Phycol..

[9] Nakai M., Kageyama N., Nakahara K., Miki W. (2006). Phlorotannins as radical scavengers from the extract of *Sargassum ringgoldianum*. Mar. Biotechnol..

[10] Duan W., Ji W., Wei Y., Zhao R., Chen Z., Geng Y., Jing F., Wang X. (2018). Separation and purification of fructo-oligosaccharide by High-Speed Counter-Current Chromatography coupled with precolumn derivatization. Molecules.

[11] Yan R., Shen J., Liu X., Zou Y., Xu X. (2018). Preparative isolation and purification of hainanmurpanin, meranzin, and phebalosin from leaves of Murraya exotica L. using supercritical fluid extraction combined with consecutive high-speed countercurrent chromatography. J. Sep. Sci..

[12] Guo W., Dong H., Wang D., Yang B., Wang X., Huang L. (2018). Separation of seven polyphenols from the rhizome of *Smilax glabra* by offline two dimension recycling HSCCC with extrusion mode. Molecules.

[13] Liu Y., Zhou X., Naman C.B., Lu Y., Ding L., He S. (2018). Preparative separation and purification of trichothecene mycotoxins from the marine fungus *Fusarium* sp. LS68 by high-speed countercurrent chromatography in stepwise elution mode. Mar. Drugs.

[14] Wang J., Gu D., Wang M., Guo X., Li H., Dong Y., Guo H., Wang Y., Fan M., Yang Y. (2017). Rational approach to solvent system selection for liquid–liquid extraction–assisted sample pretreatment in counter–current chromatography. J. Chromatogr. B.

[15] Shaheen N., Lu Y., Geng P., Shao Q., Wei Y. (2017). Isolation of four phenolic compounds from *Mangifera indica*. L flowers by using normal phase combined with elution extrusion two-step high speed countercurrent chromatography. J. Chromatogr. B.

[16] Kong Q., Ren X., Hu R., Yin X., Jiang G., Pan Y. (2016). Isolation and purification of two antioxidant isomers of resveratrol dimer from the wine grape by counter-current chromatography. J. Sep. Sci..

